# Standardized and reproducible methodology for the comprehensive and systematic assessment of surgical resection margins during breast-conserving surgery for invasive breast cancer

**DOI:** 10.1186/1471-2407-9-254

**Published:** 2009-07-27

**Authors:** Stephen P Povoski, Rafael E Jimenez, Wenle P Wang, Ronald X Xu

**Affiliations:** 1 Division of Surgical Oncology, Department of Surgery, Arthur G. James Cancer Hospital and Richard J. Solove Research Institute and Comprehensive Cancer Center, The Ohio State University, Columbus, Ohio, 43210, USA; 2 Department of Pathology, The Ohio State University, Columbus, Ohio, 43210, USA; 3 Current address : Division of Anatomic Pathology, Department of Laboratory Medicine and Pathology, Mayo Clinic, Rochester, Minnesota, 55905, USA; 4 Current address : Department of Pathology, VA Medical Center at Baltimore, Baltimore, Maryland, 21201, USA; 5 Department of Biomedical Engineering, The Ohio State University, Columbus, Ohio, 43210, USA

## Abstract

**Background:**

The primary goal of breast-conserving surgery (BCS) is to completely excise the tumor and achieve "adequate" or "negative" surgical resection margins while maintaining an acceptable level of postoperative cosmetic outcome. Nevertheless, precise determination of the adequacy of BCS has long been debated. In this regard, the aim of the current paper was to describe a standardized and reproducible methodology for comprehensive and systematic assessment of surgical resection margins during BCS.

**Methods:**

Retrospective analysis of 204 BCS procedures performed for invasive breast cancer from August 2003 to June 2007, in which patients underwent a standard BCS resection and systematic sampling of nine standardized re-resection margins (superior, superior-medial, superior-lateral, medial, lateral, inferior, inferior-medial, inferior-lateral, and deep-posterior). Multiple variables (including patient, tumor, specimen, and follow-up variables) were evaluated.

**Results:**

6.4% (13/204) of patients had positive BCS specimen margins (defined as tumor at inked edge of BCS specimen) and 4.4% (9/204) of patients had close margins (defined as tumor within 1 mm or less of inked edge but not at inked edge of BCS specimen). 11.8% (24/204) of patients had at least one re-resection margin containing additional disease, independent of the status of the BCS specimen margins. 7.1% (13/182) of patients with negative BCS specimen margins (defined as no tumor cells seen within 1 mm or less of inked edge of BCS specimen) had at least one re-resection margin containing additional disease. Thus, 54.2% (13/24) of patients with additional disease in a re-resection margin would not have been recognized by a standard BCS procedure alone (P < 0.001). The nine standardized resection margins represented only 26.8% of the volume of the BCS specimen and 32.6% of the surface area of the BCS specimen.

**Conclusion:**

Our methodology accurately assesses the adequacy of surgical resection margins for determination of which individuals may need further resection to the affected breast in order to minimize the potential risk of local recurrence while attempting to limit the volume of additional breast tissue excised, as well as to determine which individuals are not realistically amendable to BCS and instead need a completion mastectomy to successfully remove multifocal disease.

## Background

Breast-conserving surgery (BCS) followed by whole-breast radiation therapy is a well-established standard of care for the treatment of early-stage invasive breast cancer [1,2]. This has been shown to be essentially equivalent to mastectomy with regards to local control and survival [3-8]. The primary goal of BCS is to completely excise the tumor and achieve "adequate" or "negative" surgical resection margins while maintaining an acceptable level of postoperative cosmetic outcome to the affected breast. Nevertheless, the precise determination of the adequacy of surgical resection margins during BCS has long been debated [8-17]. There have been two major questions that have been central to this ongoing debate. First, what represents the most appropriate definition of an "adequate" or "negative" surgical margin? Second, what is the most optimal manner in which to assess the adequacy of the surgical margins?

In this regard, the aim of the current paper was to describe a standardized and reproducible methodology for the comprehensive and systematic assessment of surgical resection margins during BCS for invasive breast cancer. This methodology attempts to limit the volume of additional breast tissue excised, yet it attempts to optimize the determination of which individual patients may need further tissue resection from the affected breast in order to minimize the potential risk of local recurrence following BCS.

## Methods

This study protocol was approved by the Clinical Scientific Review Committee (protocol number OSU 07016) and by the Cancer Institutional Review Board (protocol number 2007C0009) of the Arthur G. James Cancer Hospital and Richard J. Solove Research Institute and Comprehensive Cancer Center of The Ohio State University.

All patients who underwent BCS for invasive breast cancer were identified from a prospectively maintained operative log of a single surgeon (SPP). Prior to August 2003, each BCS procedure was done without the use of any standardized methodology of assessing the surrounding perimeter of the resultant BCS resection bed cavity. Starting in August 2003, such a standardized and reproducible methodology of assessment of surgical resection margins (for both the BCS specimen itself and for the surrounding perimeter of the resultant BCS resection bed cavity) during BCS for invasive breast cancer was initiated by the surgeon (SPP). Therefore, all BCS cases from August 2003 to June 2007 for invasive breast cancer were selected for the current analysis. All BCS procedures performed for a diagnosis of ductal carcinoma in situ (DCIS) alone were not included in the current analysis. BCS was not offered or undertaken in patients that had any additional suspicious lesions within the affected breast that were demonstrated on preoperative mammogram, ultrasound, or magnetic resonance imaging (MRI) unless those additional suspicious lesions were first biopsied and proven not to represent additional foci of breast malignancy within the affected breast. In those cases in which those additional suspicious lesions were first biopsied and proven to represent additional foci of breast malignancy within the affected breast or in those cases in which patients elected not to have those additional suspicious lesions biopsied within the affected breast, only mastectomy was offered to those patients.

All cases were performed in the operating room at the Arthur G. James Cancer Hospital and Richard J. Solove Research Institute of The Ohio State University. These procedures were generally done under general anesthesia along with concomitant evaluation of the axillary lymph nodes with sentinel lymph node biopsy and axillary lymph node dissection, when indicated. For all non-palpable or questionably-palpable tumors, pre-resection tumor localization was accomplished by either mammographic guidance or sonographic guidance.

**Figure 1:**
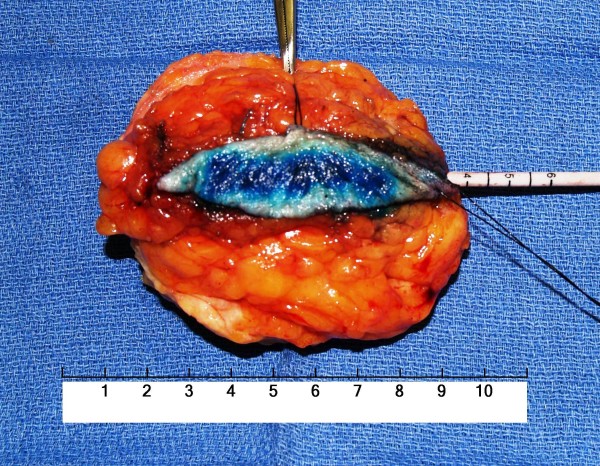
**Photograph of a typical example of a breast-conserving surgery (BCS) specimen taken from a left breast**.

A standardized rationale was consistently utilized for the determination of the extent of tissue resection during each BCS procedure. For each BCS procedure, an overlying skin ellipse was incised with a #10 surgical blade. Division of the breast parenchymal was performed with the coagulation mode of the electrocautery (Force 4B Electrosurgical Generator, Valleylab™, Boulder, Colorado). In all cases, wide local excision of breast parenchyma around the tumor was undertaken, along with en bloc removal of an ellipse of overlying skin (Figure 1). As is a standard of practice for BCS, all attempts were made by the operating surgeon to remove the tumor with what grossly appeared at the time of surgery to be at least a 1 cm rim of surrounding normal-appearing breast tissue around all aspects of the tumor. Additionally, concomitant excision of underlying breast parenchyma all the way down to and including the pectoralis major muscle fascia was performed for all tumors located within the posterior two-thirds of the breast parenchyma and was performed only selectively for tumors located within the anterior one-third of the breast parenchyma. Conversely, such a concomitant excision of breast parenchyma all the way down to and including the pectoralis major muscle fascia was generally excluded for tumors located very superficially within the breast parenchyma in a location just under the skin of the breast. Lastly, for very deeply placed tumors abutting or involving the underlying pectoralis major muscle fascia, concomitant en bloc excision of a portion of the directly underlying pectoralis major muscle was generally performed. No percentage criteria for the minimum required amount of breast tissue that was necessary to be resected from the affected breast was used in this rationale for the extent of the BCS procedure, but rather this rationale was based upon the determination of the appropriateness of the extent of resection, and when determined necessary, to include all layers comprising the field of the affected breast (i.e., skin, breast parenchyma, pectoralis major muscle fascia, and pectoralis major muscle) in order to maximize the likelihood of surgical resection margin clearance. Each BCS specimen (Figure 1) was marked with black silk sutures on the skin ellipse for specimen orientation. This generally consisted of a long black silk suture marking the lateral edge of the skin ellipse and a short black silk suture marking the superior edge of the skin ellipse.

**Figure 2:**
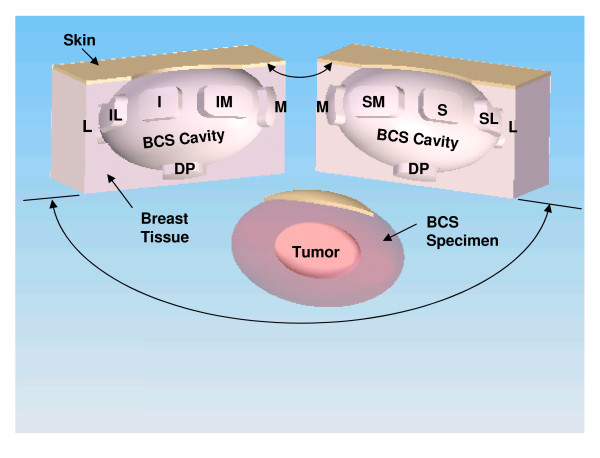
**Computer-generated representation of the resultant breast-conserving surgery (BCS) resection bed cavity and the BCS specimen resulting from a BCS procedure performed on a left breast**. In this example, the area of the BCS resection bed cavity has been bisected along its long axis to illustrate the exact spatial location from where the nine standardized re-resection margins were sampled from the superior (S), superior-medial (SM), superior-lateral (SL), medial (M), lateral (L), inferior (I), inferior-medial (IM), inferior-lateral (IL), and deep-posterior (DP) aspects of the BCS resection bed cavity.

**Figure 3:**
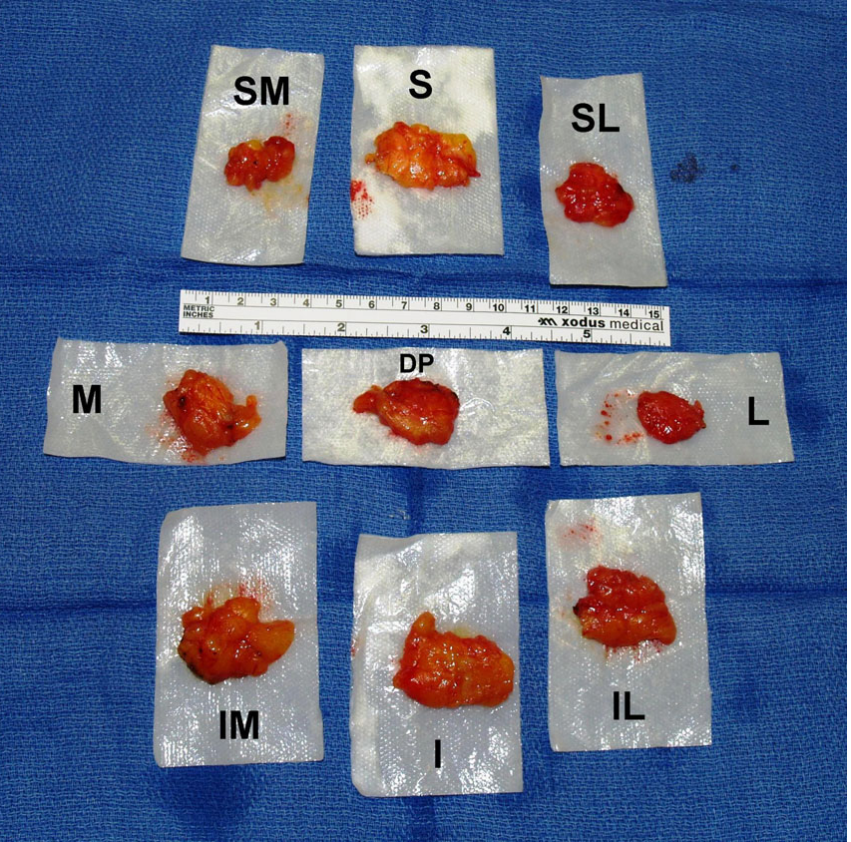
**Photograph of a typical example of the nine standardized re-resection margins sampled from the superior (S), superior-medial (SM), superior-lateral (SL), medial (M), lateral (L), inferior (I), inferior-medial (IM), inferior-lateral (IL), and deep-posterior (DP) aspects of a left-sided breast-conserving surgery (BCS) resection bed cavity**.

Once the BCS specimen was removed, rectangular-shaped pieces of breast tissue were then systematically sampled from nine standardized locations within the perimeter of the resultant BCS resection bed cavity using curved Mayo scissors and/or a #24 surgical blade (Figure 2). These rectangular-shaped pieces of breast tissue were excised with the curved Mayo scissors and/or a #24 surgical blade, instead of with the coagulation mode of the electrocautery, in order to generate no evidence of cautery artifact on that particular surface of each sampled piece of breast tissue. Therefore, in the end, the non-cauterized aspect of each rectangular-shaped piece of breast tissue sampled would represent the surface of the rectangular-shaped piece of breast tissue that was furthest from the corresponding resection margin of the originally submitted BCS specimen that was removed from the affected breast with the coagulation mode of the electrocautery and the cauterized aspect of each rectangular-shaped piece of breast tissue sampled would represent the surface of the rectangular-shaped piece of breast tissue that was abutting the corresponding resection margin of the originally submitted BCS specimen that was removed from the affected breast with the coagulation mode of the electrocautery. In this fashion, the distribution of electrocautery artifact on each rectangular-shaped piece of breast tissue sampled was used by the pathologist to help distinguish the actual true margin surface (non-cauterized surface) from that of the non-margin surface (cauterized surface) of each rectangular-shaped piece of breast tissue sampled. These rectangular-shaped pieces of breast tissue that where systematically sampled from nine standardized locations within the perimeter of the resultant BCS resection bed cavity were designated as originating from the superior, superior-medial, superior-lateral, medial, lateral, inferior, inferior-medial, inferior-lateral, and deep-posterior aspects of the resultant BCS resection bed cavity (Figures 2 and 3). These rectangular-shaped pieces of breast tissue that were systematically sampled from nine standardized locations within the perimeter of the resultant BCS resection bed cavity were designated as the nine standardized re-resection margin specimens, as they are referred to throughout the remainder of the current paper. Since the anterior aspect of the BCS specimen was covered by an overlying skin ellipse (in all but two cases), there was no anterior re-resection margin designation for the resultant BCS resection bed cavity.

Intraoperative sectioning and subsequent intraoperative gross assessment of BCS margins on the BCS specimens were not undertaken by the operating surgeon within the operating room for any of the 204 BCS cases. This was done in this fashion at the request of the Surgical Pathology Department in order to maintain the integrity of the intact BCS specimen for standard tissue processing that would be subsequently performed in the Surgical Pathology Department. However, in appropriately selected cases, verification of the relationship of the tumor, marking clip, and/or biopsy cavity to the edges of the BCS specimen was evaluated on non-compression specimen mammography and/or on intraoperative specimen sonography of the intact BCS specimen.

Both the BCS specimen and the nine standardized re-resection margin specimens for each BCS case were sent to the Surgical Pathology Department for standard tissue processing. The three-dimensional measurements (length, height, and width) of each submitted specimen were recorded.

After standard tissue processing, each BCS specimen was inked in a color-specific fashion using multiple colored inks to designate the superior, medial, lateral, inferior, deep-posterior, and anterior-skin surfaces. Each BCS specimen was then grossly serially sectioned (at a thickness of approximately 5 mm for each serial section and in an anterior skin surface to posterior direction) along the entire long axis length of the BCS specimen (i.e., by a standard perpendicular margins/radial margins technique). The macroscopic three-dimensional measurements (length, height, and width) of the tumor, residual tumor, or previous diagnostic excisional biopsy cavity identified within each BCS specimen were recorded, as well as the microscopic distance of the tumor, residual tumor, or previous diagnostic excisional biopsy cavity from the superior, medial, lateral, inferior, deep-posterior, and anterior-skin inked surfaces of the BCS specimen. Along the periphery of each BCS specimen, six standardized locations were sampled as BCS specimen margins, and were designated as the superior, medial, lateral, inferior, deep-posterior, and anterior-skin margins of the BCS specimen. All submitted BCS specimen tissue sections were processed for permanent histopathologic evaluation with routine Hematoxylin and Eosin (H&E) staining. No intraoperative frozen section analysis was undertaken for microscopic evaluation of the BCS specimen margins. The six designated BCS specimen margin locations along the periphery of each BCS specimen were microscopically assessed at the time of permanent histopathologic evaluation for the presence or absence of invasive carcinoma or DCIS. The six designated BCS specimen margin locations along the periphery of each BCS specimen were then each defined as a "positive" BCS specimen margin, a "close" BCS specimen margin, or a "negative" BCS specimen margin, based upon the finding of invasive carcinoma or DCIS at the six designated BCS specimen margin locations. A "positive" BCS specimen margin was defined as tumor cells seen at the inked edge of the BCS specimen [3,8]. A "close" BCS specimen margin was defined as tumor cells seen within 1 mm or less of the inked edge of the BCS specimen, but no tumor cells seen at the inked edge of the BCS specimen. A "negative" BCS specimen margin was defined as no tumor cells seen within 1 mm or less of the inked edge of the BCS specimen. For the six designated BCS specimen margin locations along the periphery of each BCS specimen, the exact distance to the closest BCS specimen margin was determined. However, for those individuals who had undergone a previous surgical (excisional) biopsy as their initial diagnostic breast biopsy and had no residual disease within the BCS specimen, the exact distance to the closest BCS specimen margin could not be determined. For all cases in which BCS specimen margins were reported generically as "close" (and not reported as an exact numerical measurement) in the original official pathology report, repeat microscopic histopathologic evaluation was subsequently performed by a pathologist (REJ) to more precisely determine the exact numerical measurement of the distance between tumor and the inked edge of the BCS specimen. The presence of only LCIS, atypical lobular hyperplasia, or atypical ductal hyperplasia within any one of the six designated BCS specimen margin locations along the periphery of each BCS specimen was not considered a "positive" finding for margin definition purposes.

The nine standardized re-resection margin specimens collected on each BCS case were inked with black ink, serially sectioned, and submitted in entirety for processing for permanent histopathologic evaluation with routine H&E staining. No intraoperative frozen section analysis was undertaken for intraoperative microscopic evaluation of any of the nine standardized re-resection margin specimens. All nine standardized re-resection margin specimens were assessed for the presence ("positive") or absence ("negative") of invasive carcinoma or DCIS. The presence of only lobular carcinoma in situ, atypical lobular hyperplasia, or atypical ductal hyperplasia within a standardized re-resection margin specimen was not considered a "positive" finding. The electrocautery artifact on each of the nine standardized re-resection margin specimens was specifically used by the pathologist to evaluate the relationship any focus of invasive carcinoma or DCIS within each standardized re-resection margin specimen to that of its actual margin surface (non-cauterized surface) and its non-margin surface (cauterized surface).

The concordance and disconcordance of the spatial location of a positive re-resection margin specimen in comparison to the margin status of the corresponding BCS specimen was assessed for all cases in which there was at least one positive re-resection margin among the nine submitted standardized re-resection margin specimens. When both a BCS specimen margin was defined as a "positive" margin or as a "close" margin and one or more of the standardized re-resection margin specimens was defined as "positive", the spatial location of these findings were evaluated to assess concordance versus disconcordance of these findings. A concordant finding was defined as having an exact-matched finding within the same-named location or a near-matched finding within the same-named location for a BCS case having both a "positive" margin or a "close" margin on the BCS specimen at a specifically defined margin location (i.e., superior, medial, lateral, inferior, or deep-posterior) and a "positive" standardized re-resection margin specimen of the corresponding standardized re-resection specimen location (i.e., superior, superior-medial, superior-lateral, medial, lateral, inferior, inferior-medial, inferior-lateral, or deep-posterior). In this instance, an exact-matched finding was defined as being within the same-named location for the BCS specimen margin location (as pertaining only to the superior, medial, lateral, inferior, and deep-posterior aspects) and for the standardized re-resection margin specimen location (as pertaining only to the superior, medial, lateral, inferior, and deep-posterior aspects). In this instance, a near-matched finding was defined as being within one adjacent location for the BCS specimen margin location (as pertaining only to the superior, medial, lateral, and inferior aspects) and for the standardized re-resection margin specimen location (as pertaining only to the superior, superior-medial, superior-lateral, medial, lateral, inferior, inferior-medial, and inferior-lateral aspects). A disconcordant finding was defined as having a non-matched finding of a "positive" margin or a "close" margin on the BCS specimen at a specifically defined margin location as compared to the "positive" standardized re-resection margin location. In this instance, a non-matched finding was defined as being at a spatial distance of more than one adjacent location away from one another for the BCS specimen margin locations (as pertaining only to the superior, medial, lateral, and inferior aspects) and for the standardized re-resection margin specimen locations (pertaining only to the superior, superior-medial, superior-lateral, medial, lateral, inferior, inferior-medial, and inferior-lateral aspects). Additionally, we utilized the term disconcordant finding to define a BCS case in which there were "negative" BCS margins while having one or more "positive" standardized re-resection margin locations. Lastly, in those instances in which there were multiple "positive" findings for a given BCS case, the classification could be expanded to include concordant findings, disconcordant findings, or both simultaneously concordant and disconcordant findings.

The histopathologic findings from the official, finalized pathology report for each BCS case was discussed in person with each patient at the one-week postoperative follow-up visit. The potential clinical significance and implications of any abnormal findings seen on histopathologic evaluation (i.e., concern of a compromised surgical margin of resection as defined by a positive BCS specimen margin, a close BCS specimen margin, and/or a positive re-resection margin) were explained to each patient. All scenarios, in which further surgical intervention to the affected breast was thought to be of potential benefit, were fully disclosed to and discussed with each patient and those scenarios were appropriately managed in those patients who accepted such further surgical intervention to the affected breast.

Patients were referred to radiation oncology for consideration of standard postoperative whole-breast radiation therapy to the affected breast as part of their breast-conserving therapy. Additionally, patients were referred to medical oncology for consideration of postoperative adjuvant therapy, when appropriate. All attempts were made to maintain ongoing, serial patient follow-up with all patients. However, some patients have remained noncompliant and have not maintained ongoing, serial follow-up, even after multiple attempts to arrange such follow-up for these noncompliant patients.

The volume of each BCS specimen was calculated using the formula of the volume of an ellipsoid [4/3·π·length axis radius·width axis radius·height axis radius], rather than using the formula of the volume of a cuboid [length·width·height], since the three-dimensional shape of any given BCS specimen generally better approximated that of an ellipsoid rather than that of a cuboid. The volume of the nine standardized re-resection margin specimens was calculated using the formula of the volume of a cuboid, rather than the formula of the volume of an ellipsoid, since each standardized re-resection margin specimen generally better approximated that of a cuboid rather than that of an ellipsoid. The three-dimensional surface area of each BCS specimen was calculated using the derived formula of the surface area of an ellipsoid [18,19]. Due to the significant complexity of the formula used to calculate the surface area of an ellipsoid, the derivation of that formula will not be shown within the text of the current paper. The computer program MATLAB^® ^(The MathWorks, Inc., Natick, Massachusetts) was used to assist in the calculation the surface area of an ellipsoid from the three-dimensional measurements (length, height, and width) of each BCS specimen. The two-dimensional surface area of the face of each standardized re-resection margin specimen, based on the two largest dimensions of each standardized re-resection margin specimen (i.e., length and width), was calculated simply using the formula of the two-dimensional area of a rectangle [length·width]. The following percentage were then calculated: (1) the percentage of the cumulative volume of all nine standardized re-resection margins as compared to each BCS specimen volume, (2) the percentage of cumulative 2-dimensional surface area of all nine standardized re-resection margins as compared to the surface area of each BCS specimen, and (3) the percentage of the cumulative 2-dimensional surface area of all nine standardized re-resection margins as compared to the surface area of each BCS specimen minus the surface area of the overlying skin ellipse.

For this study, data collection of all variables analyzed was accomplished by way of retrospective review of the electronic medical records system of The Ohio State University Medical Center. Multiple variables were assessed and evaluated, including patient demographics, tumor variables, specimen variables, and patient follow-up variables. The software program SPSS^® ^16.0 for Windows^® ^(SPSS, Inc., Chicago, Illinois) was used for the data analyses of all variables captured during data collection. For univariate comparisons of categorical variables, either Pearson chi-square test or Fisher exact test was utilized. For univariate comparisons of continuous variables, one-way analysis of variance (ANOVA) was utilized. All reported univariate P-values were two-sided. All univariate P-values determined to be 0.05 or less were considered to be significant. Multivariate logistic regression analysis was performed in appropriately selected situations on all variables with a univariate P-value of 0.10 or less, in order to assess for the determination of possible independent predictors.

## Results

From August 2003 to June 2007, 204 BCS procedures were performed for invasive breast cancer by a single surgeon (SPP). Patient demographics and tumor characteristics for all 204 BCS cases are shown in Table 1 and Table 2, respectively. Of the 204 BCS procedures, 164 were performed as BCS procedures in patients with an intact tumor who had undergone only a minimally-invasive diagnostic breast biopsy at the time of initial diagnosis and 40 were performed as re-excision BCS procedures in patients who had previously undergone a surgical (excisional) biopsy as their initial diagnostic breast biopsy. Measurement variables for the BCS specimens are shown in Table 3. Measurement variables for the nine standardized re-resection margin specimens are shown in Table 4. The percentage of the cumulative volume of all nine standardized re-resection margins as compared to each BCS specimen volume, the percentage of cumulative 2-dimensional surface area of all nine standardized re-resection margins as compared to the surface area of each BCS specimen, and the percentage of the cumulative 2-dimensional surface area of all nine standardized re-resection margins as compared to the surface area of each BCS specimen minus the surface area of the overlying skin ellipse are shown in Table 5.

**Table 1:** Patient demographics of all 204 BCS cases

Patient demographics	Number (percentage or range)
Age (years)	57 (27 – 87)
Height (inches)	65 (54 – 72)
Weight (pounds)	175 (96 – 342)
Body mass index	29.6 (16.6 – 58.7)
	
Race	
Caucasian	175 (86%)
Black	29 (14%)
	
Menopausal status	
Postmenopausal	154 (76%)
Premenopausal	43 (21%)
Perimenopausal	7 (3%)
	
Breast sidedness	
Right	98 (48%)
Left	106 (52%)
	
Breast size	
Small (approximately A-cup)	17 (8%)
Medium (approximately B-cup)	82 (40%)
Large (approximately C-cup)	76 (37%)
Extra-large (approximately D-cup or greater)	29 (14%)
	
Neoadjuvant therapy	30 (14.7%)
Neoadjuvant systemic chemotherapy	24 (11.8%)
Neoadjuvant anti-estrogen therapy	6 (2.9%)

BCS, breast-conserving surgery

**Table 2:** Tumor characteristics of all 204 BCS cases

Tumor characteristics	Number (percentage or range)
Originally palpable mass	
Yes	79 (39%)
No	112 (55%)
Indeterminate	13 (6%)
	
Tumor (pT) size (centimeters)	1.6 (0.08 – 6.0)
	
Tumor location	
	
Upper outer quadrant	105 (52%)
Upper inner quadrant	40 (20%)
Lower outer quadrant	29 (14%)
Lower inner quadrant	22 (11%)
Subareolar	8 (4%)
	
Tumor histopathology	
Invasive ductal carcinoma	165 (81%)
Invasive lobular carcinoma	12 (6%)
Mixed invasive ductal and lobular carcinoma	8 (4%)
Invasive carcinoma not otherwise specified	7 (3%)
Mucinous (colloid) carcinoma	7 (3%)
Papillary carcinoma	3 (2%)
Adenoid cystic carcinoma	2 (1%)
	
Histologic grade	
Well-differentiated	52 (25%)
Moderately-differentiated	71 (35%)
Poorly-differentiated	81 (40%)
	
Estrogen receptor positive	155 (76%)
	
Progesterone receptor positive	135 (66%)
	
Her 2 Neu positive	26 (12.7%)
	
Lymphovascular invasion	36 (18%)
	
Associated ductal carcinoma in situ (DCIS)	131 (64%)
	
Associated axillary lymph node involvement	
Yes	37 (18%)
No	158 (78%)
Not assessed	9 (4%)
	
Type of original diagnostic breast biopsy	
14-gauge core biopsy	74 (36%)
8- or 11-gauge ultrasound mammotome biopsy	52 (25%)
8- or 11-gauge stereotactic mammotome biopsy	38 (19%)
Surgical (excisional) biopsy	40 (20%)

BCS, breast-conserving surgery

**Table 3:** Summary of BCS specimen variables from the pathology report of the 204 BCS cases

BCS specimen variables	Mean measurement (range)
Dimension 1: Length (cm)	8.5 (4.4 – 18.7)
Dimension 2: Width (cm)	6.8 (2.5 – 12.8)
Dimension 3: Height (cm)	3.1 (2.0 – 7.0)
Volume of BCS specimen (cm^3^)	103.3 (12.0 – 538.9)
Surface area of BCS specimen (cm^2^)	122.5 (28.9 – 437.9)
Surface area of overlying skin ellipse (cm^2^)	8.0 (0 – 47.1)
Surface area of BCS specimen minus overlying skin ellipse (cm^2^)	113.7 (21.2 – 421.7)
Weight (gm)	86.9 (15 – 330)#

BCS, breast-conserving surgery

#: The BCS specimen weight was available for only 162 of the 204 BCS cases.

**Table 4:** Summary of the nine standardized re-resection margin variables from the pathology report of the 204 BCS cases

Re-resection margin variables	Mean measurement (range)
Superior (S) re-resection margin	
Volume (cm^3^)	2.74 (0.17 – 19.24)
2-dimensional surface area (cm^2^)	4.23 (0.56 – 23.20)
	
Superior-Medial (SM) re-resection margin	
Volume (cm^3^)	2.60 (0.04 – 50.11)
2-dimensional surface area (cm^2^)	3.77 (0.20 – 28.50)
	
Superior-Lateral (SL) re-resection margin	
Volume (cm^3^)	2.44 (0.14 – 52.73)
2-dimensional surface area (cm^2^)	3.85 (0.50 – 28.40)
	
Medial (M) re-resection margin	
Volume (cm^3^)	3.09 (0.05 – 36.00)
2-dimensional surface area (cm^2^)	4.50 (0.20 – 36.00)
	
Lateral (L) re-resection margin	
Volume (cm^3^)	2.69 (0.16 – 15.12)
2-dimensional surface area (cm^2^)	4.12 (0.80 – 21.60)
	
Inferior (I) re-resection margin	
Volume (cm^3^)	3.04 (0.10 – 24.57)
2-dimensional surface area (cm^2^)	4.66 (0.50 – 18.90)
	
Inferior-Medial (IM) re-resection margin	
Volume (cm^3^)	2.33 (0.10 – 31.50)
2-dimensional surface area (cm^2^)	3.80 (0.30 – 15.80)
	
Inferior-Lateral (IL) re-resection margin	
Volume (cm^3^)	2.33 (0.10 – 10.10)
2-dimensional surface area (cm^2^)	4.05 (0.50 – 20.30)
	
Deep-Posterior (DP) re-resection margin	
Volume (cm^3^)	3.29 (0.10 – 66.00)
2-dimensional surface area (cm^2^)	4.67 (0.10 – 33.00)
	
Cumulative volume (cm^3^) of all nine standardized re-resection margins	24.55 (2.20 – 135–57)
	
Cumulative 2-dimensional surface area (cm^2^) of all nine standardized re-resection margins	37.66 (6.19 – 102.66)

BCS, breast-conserving surgery

**Table 5:** Summary of percentage of cumulative volume of all nine standardized re-resection margins as compared to the BCS specimen volume, percentage of cumulative 2-dimensional surface area of all nine standardized re-resection margins as compared to the surface area of the BCS specimen, and percentage of cumulative 2-dimensional surface area of all nine standardized re-resection margins as compared to the surface area of the BCS specimen minus the surface area of the overlying skin ellipse from the pathology report of the 204 BCS cases

Variables	Percentage (range)
Percentage of cumulative volume of all nine standardized re-resection margins as compared to the BCS specimen volume	26.8% (3.5% – 85.7%)
Percentage of cumulative 2-dimensional surface area of all nine standardized re-resection margins as compared to the surface area of the BCS specimen	32.6% (7.5% – 96.3%)
Percentage of cumulative 2-dimensional surface area of all nine standardized re-resection margins as compared to the surface area of the BCS specimen	
minus the surface area of the overlying skin ellipse	34.9% (8.1% – 98.2%)

BCS, breast-conserving surgery

A description of the margin status of the BCS specimen and the re-resection margin status of the nine standardized re-resection margin specimens at the time of the original BCS procedure for all 204 BCS cases is shown in Table 6. Likewise, a description of the frequency of BCS cases undergoing a subsequent re-excision breast procedure and the frequency of BCS cases in which residual disease was found within those subsequent re-excision breast procedure specimens is shown in Table 6.

**Table 6:** Description of the margin status of the BCS specimen and the re-resection margin status of the nine standardized re-resection margin specimens at the time of the original BCS procedure for all 204 BCS cases, as well as the description of the frequency of patients undergoing a subsequent re-excision breast procedure and the frequency of finding residual disease within those subsequent re-excision breast procedure specimens

Description of margin variables for original BCS procedure	Status of margin variables for original BCS specimen	Frequency of a subsequent re-excision	Frequency of residual disease
Positive BCS specimen margin(s)	13/204 (6.4%)	7/13 (53.8%)^#,£^	2/7 (28.6%)
Close BCS specimen margin(s)	9/204 (4.4%)	4/9 (44.4%)^¶^	2/4 (50.0%)
Positive or close BCS specimen margin(s)	22/204 (10.8%)	11/22 (50.0%)^Ω ,£^	4/11 (36.4%)
Negative BCS specimen margins	182/204 (89.2%)	0/182 (0%)	not applicable
Positive re-resection margin(s)	24/204 (11.8%)	15/24 (62.5%)* ^,±, Ø, ♀^	4/15 (26.7%)
Positive BCS specimen margin(s) AND positive re-resection margin(s)	8/204 (3.9%)	7/8 (87.5%)^£^	2/7 (28.6%)
Close BCS specimen margin(s) AND positive re-resection margin(s)	3/204 (1.5%)	3/3 (100%)	1/3 (33.3%)
Positive or close BCS specimen margin(s) AND positive re-resection margin(s)	11/204 (5.4)	10/11 (90.9%)^£^	3/10 (30.0%)
Positive BCS specimen margin(s) AND negative re-resection margins	5/204 (2.5%)	0/5 (0%)^#^	not applicable
Close BCS specimen margin(s) AND negative re-resection margins	6/204 (2.9%)	1/6 (16.7%)^¶^	1/1 (100%)
Positive or close BCS specimen margin(s) AND negative re-resection margins	11/204 (5.4%)	1/11 (9.1%)^Ω^	1/1 (100%)
Negative BCS specimen margins AND positive re-resection margin(s)	13/204 (6.4%)^@^	5/13 (38.5%)* ^,±^	0/5 (0%)
Negative BCS specimen margins AND negative re-resection margins	169/204 (82.8%)	0/169 (0%)	not applicable
Positive BCS specimen margin(s) AND/OR close BCS specimen margin(s) AND/OR positive re-resection margin(s)	35/204 (17.2%)	16/35 (45.7%)^Ω^, *, ^±, Ø^	5/16 (31.3%)

BCS, breast-conserving surgery

#: Five patients with a positive BCS specimen margin declined a subsequent re-excision procedure due to the finding of nine negative standardized re-resection margins.

¶: Five patients with a close BCS specimen margin declined a subsequent re-excision procedure due to the finding of nine negative standardized re-resection margins.

£: One patient refused the recommendation of the surgeon for the absolute need of a subsequent re-excision procedure.

Ω: Ten patients with a positive or close BCS specimen margin declined a subsequent re-excision procedure due to the finding of nine negative standardized re-resection margins.

*: Five patients declined a subsequent re-excision procedure due to the finding of one isolated positive standardized re-resection margin out of nine standardized re-resection margins in which the noncauterized surface (i.e., actual true margin surface) of that standardized re-resection margin was free of tumor and therefore was considered a microscopically negative final margin.

±: Three patients refused the recommendation of the surgeon for the absolute need of a subsequent re-excision procedure.

Ø: One patient declined a subsequent re-excision procedure due to the finding of in situ carcinoma within one standardized re-resection margin for which it could not be distinguished as to whether it represented lobular carcinoma in situ versus ductal carcinoma in situ.

♀: Eight of nine patients with multiple positive standardized re-resection margins agreed to a subsequent re-excision procedure.

@: 7.1% (13/182) of patients with negative BCS specimen margins had at least one re-resection margin containing additional tumor.

Among all 204 BCS cases, 13 (6.4%) had a positive BCS specimen margin and 9 (4.4%) had a close BCS specimen margin, thus giving a total of 22 (10.8%) cases having either a positive or close BCS specimen margin (Table 6). Of those 13 cases in which there was a positive BCS specimen margin, eight had one positive BCS specimen margin, three had two positive BCS specimen margins, and two had three positive BCS specimen margins, thus representing 20 total positive BCS specimen margins among a total of 1224 BCS specimen margin locations evaluated for the 204 BCS cases. Of those 22 cases in which there was a positive or close BCS specimen margin, 17 had one positive/close BCS specimen margin, three had two positive/close BCS specimen margins, and two had three positive/close BCS specimen margins, thus representing 29 total positive/close BCS specimen margins among a total of 1224 BCS specimen margin locations evaluated for the 204 BCS cases. A summary of the findings for all the positive BCS specimen margin cases and the positive or close BCS specimen margin cases is shown in Table 7.

**Table 7:** Summary of the total number of individual instances in which any one of the designated BCS specimen margin sites was positive or was positive/close from the pathology report of the 204 BCS cases

BCS specimen margin	Number of instances in which there was a positive BCS specimen margin	Histopathology of each positive BCS specimen margin
Superior (S) margin	3	2 IDC, 1 ACC
Medial (M) margin	5	2 IDC, 2 ILC, 1 DCIS
Lateral (L) margin	3	1 IDC, 1 ILC, 1 DCIS
Inferior (I) margin	5	2 IDC, 2 ILC, 1 DCIS
Deep-Posterior (DP) margin	4	2 IDC, 1 ILC, 1 ACC
Anterior-Skin (AS) margin	0	not applicable

BCS specimen margin	Number of instances in which there was a positive/close BCS specimen margin	Histopathology of each positive/close BCS specimen margin

Superior (S) margin	3	2 IDC, 1 ACC
Medial (M) margin	8	5 IDC, 2 ILC, 1 DCIS
Lateral (L) margin	4	2 IDC, 1 ILC, 1 DCIS
Inferior (I) margin	6	2 IDC, 2 ILC, 2 DCIS
Deep-Posterior (DP) margin	8	4 IDC, 1 ILC, 2 DCIS, 1 ACC
Anterior-Skin (AS) margin	0	not applicable

BCS, breast-conserving surgery; IDC, invasive ductal carcinoma; ILC, invasive lobular carcinoma; DCIS ductal carcinoma in situ; ACC, adenoid cystic carcinoma

For the 164 patients that had an intact tumor at the time of the BCS procedure, the mean microscopic distance of tumor to the closest BCS specimen margin was 0.70 cm (range, 0 to 2.00 cm). Additionally, for those 164 patients that had an intact tumor at the time of the BCS procedure, the mean microscopic distance of tumor to the closest BCS specimen margin was 0.79 cm (range, 0.16 to 2.00 cm) for those classified as having a negative BCS specimen margin (n = 146) as compared to 0.05 cm (range, 0.01 to 0.09) for those classified as having a close BCS specimen margin (n = 7) (P < 0.001).

Among the 204 BCS cases, 24 had a positive re-resection margin for one or more of the nine standardized re-resection margin specimens (Table 6). Of those 24 cases in which there was a positive re-resection margin, 15 had one positive re-resection margin, two had two positive re-resection margins, five had three positive re-resection margins, one had four positive re-resection margins, and one had five positive re-resection margins, thus representing 43 total positive re-resection margins among a total of 1836 re-resection margin specimens for the 204 BCS cases. A summary of the findings for all the positive re-resection specimens is show in Table 8. All patients with multiple positive re-resection margins accepted such further surgical intervention to the affected breast, except for one patient who refused further surgical intervention to the affected breast, as well as refused consideration of any other form of adjuvant therapy.

**Table 8:** Summary of the total number of individual instances in which any one of the designated nine standardized re-resection margin sites was positive for disease from the pathology report of the 204 BCS cases

Re-resection margin	Number of instances in which there was a positive re-resections margin	Histopathology of each positive re-resection margin
Superior (S) re-resection margin	5	2 IDC, 2 DCIS, 1 ACC
Superior-Medial (SM) re-resection margin	4	1 IDC, 2 DCIS, 1 ACC
Superior-Lateral (SL) re-resection margin	4	1 IDC, 1 ILC, 2 DCIS
Medial (M) re-resection margin	5	3 IDC, 2 DCIS
Lateral (L) re-resection margin	5	3 IDC, 2 DCIS
Inferior (I) re-resection margin	7	2 IDC, 5 DCIS
Inferior-Medial (IM) re-resection margin	3	2 IDC, 1 DCIS
Inferior-Lateral (IL) re-resection margin	7	3 IDC, 4 DCIS
Deep-Posterior (DP) re-resection margin	3	2 DCIS, 1 ACC

BCS, breast-conserving surgery; IDC, invasive ductal carcinoma; ILC, invasive lobular carcinoma; DCIS ductal carcinoma in situ; ACC, adenoid cystic carcinoma

The concordance and disconcordance of the spatial location of a positive re-resection margin specimen in comparison to the margin status of the BCS specimen was assessed for all 24 cases in which there was at least one positive re-resection margin (Table 9) and was assessed for all 11 cases in which the BCS specimen had at least one positive/close margin and there was also a positive re-resection margin (Table 10). As is shown in Table 9, of the 24 cases that had at least one positive re-resection margin, only 5 of 24 (20.8%) had completely concordant findings with the margin status of the BCS specimen, while 15 of 24 (62.5%) had completely disconcordant findings and 19 of 24 (79.2%) had some degree of disconcordant findings with the margin status of the BCS specimen. As is shown in Table 10, of the 11 patients that had at least one positive/close margin and at least one positive re-resection margin, only 5 of 11 (45.5%) had completely concordant findings between the positive/close BCS specimen margin status and the positive re-resection margin status, while 2 of 11 (18.2%) had completely disconcordant findings and 6 of 11 (54.5%) had some degree of disconcordant findings between the positive/close BCS specimen margin status and the positive re-resection margin status.

**Table 9:** Concordance or disconcordance of the spatial location of a positive re-resection margin specimen in comparison to the margin status of the corresponding spatial location on each BCS specimen for each of the 24 cases in which there as at least one positive re-resection margin specimen

Description of margin variables and concordance/disconcordance	Frequency
Negative BCS specimen margins AND positive re-resection margins (disconcordant findings)	13/24 (54.2%)
Positive/close BCS specimen margins AND positive re-resection margins (concordant findings)	5/24 (20.8%)
Positive/close BCS specimen margins AND positive re-resection margins (disconcordant findings)	2/24 (8.3%)
Positive/close BCS specimen margins AND positive re-resection margins (both concordant and disconcordant findings)	4/24 (16.7%)

BCS, breast-conserving surgery

**Table 10:** Concordance or disconcordance of the spatial location of a positive re-resection margin specimen in comparison to the margin status of the corresponding spatial location on each of the 11 cases in which the BCS specimen had at least one positive/close margin and there was also a positive re-resection margin

Description of margin variables and concordance/disconcordance	Frequency
Positive/close BCS specimen margins AND positive re-resection margins (concordant findings)	5/11 (45.5%)
Positive/close BCS specimen margins AND positive re-resection margins (disconcordant findings)	2/11 (18.2%)
Positive/close BCS specimen margins AND positive re-resection margins (both concordant and disconcordant findings)	4/11 (36.4%)

BCS, breast-conserving surgery

As shown in Table 11, any potential association of the patient demographic variables and tumor characteristic variables from Table 1 and Table 2 with a "positive final margin status" was assessed by both univariate and multivariate analyses. For the purpose of these analyses, we broadly defined a "positive final margin status" as having either a positive BCS specimen margin, a close BCS specimen margin, or an involved (positive) re-resection margin specimen. On univariate analysis, associated DCIS (P = 0.032) and larger tumor size (P = 0.034) were associated with a positive final margin status, whereas invasive lobular carcinoma displayed only a nonsignificant trend (P = 0.095). On multivariate analysis, associated DCIS (P = 0.015) and invasive lobular carcinoma (P = 0.052) were associated with a positive final margin status, whereas larger tumor size displayed only a nonsignificant trend (P = 0.066). Thus, associated DCIS was the only variable associated with a "positive final margin status" by both univariate and multivariate analyses.

**Table 11:** Potential association of patient and tumor variables with a positive final margin status (i.e., as defined as either having a positive margin, a close margin, or a positive re-resection margin) as determined by univariate and multivariate analyses

Patient and tumor variables	Univariate P-value	Multivariate P-value
Age	0.601	NA
Height	0.469	NA
Weight	0.977	NA
Body mass index	0.799	NA
Race	0.792	NA
Menopausal status	0.777	NA
Breast sidedness	0.296	NA
Breast size	0.771	NA
Neoadjuvant therapy	0.655	NA
Originally palpable mass	0.647	NA
Tumor size	0.034	0.066
Tumor location	0.411	NA
Tumor histopathology (invasive lobular carcinoma)	0.095	0.052
Histologic grade	0.727	NA
Estrogen receptor positive	0.541	NA
Progesterone receptor positive	0.132	NA
Her 2 Neu positive	0.473	NA
Lymphovascular invasion	0.374	NA
Associated DCIS	0.032	0.015
Associated axillary lymph node involvement	0.182	NA
Type of original diagnostic breast biopsy	0.430	NA

DCIS, ductal carcinoma in situ

A description of the frequency of cases undergoing a subsequent re-excision breast procedure and the frequency of cases in which residual disease was found within those subsequent re-excision breast procedure specimens is also shown in Table 6. Of those 13 cases in which there was a positive BCS specimen margin, 7 (53.8%) elected to undergo a further re-resection breast procedure, including five undergoing a re-excision BCS procedure, one undergoing a completion mastectomy, and one undergoing a re-excision BCS procedure (with further positive margins) followed by a completion mastectomy. Of those 22 cases in which there was a positive or close BCS specimen margin, 11 (50.0%) elected to undergo a further re-excision breast procedure, including six undergoing a re-excision BCS procedure, four undergoing a completion mastectomy, and one undergoing a re-excision BCS procedure (with further positive margins) followed by a completion mastectomy. Of those 35 cases in which there was a positive BCS specimen margin, a close BCS specimen margin, or a positive re-resection margin specimen, 16 (45.7%) elected to undergo a further re-excision breast procedure, including seven undergoing a re-excision BCS procedure, eight undergoing a completion mastectomy, and one undergoing a re-excision BCS procedure (with further positive margins) followed by a completion mastectomy.

Of the 204 BCS cases, 174 patients have maintained serial follow-up (with a mean follow-up duration of 38.6 months and a range of follow-up duration from 19.1 to 63.4 months), 22 patients have been noncompliant and have not maintained any identifiable follow-up, and eight patients are currently known to be deceased. Only three documented recurrences of disease related to their breast cancer are known within this series. This includes: (1) a case of recurrence within multiple distant sites in an individual undergoing a BCS procedure with an intact tumor (with positive BCS margins) who declined initial evaluation of the axillary lymph nodes, declined a subsequent recommended re-excision BCS procedure or completion mastectomy, and declined other recommended adjuvant therapies (with recurrence 4 months after the original BCS procedure); (2) a case of concurrent recurrence within the completion mastectomy site and multiple distant sites in an individual undergoing a re-excision BCS procedure (with positive BCS margins) and evaluation of the axillary lymph nodes and a subsequent completion mastectomy (with recurrence 7.5 and 6.5 months, respectively, after the original re-excision BCS procedure and subsequent completion mastectomy); and (3) a case of concurrent recurrence within the re-excision BCS site and multiple osseous metastases in an individual undergoing a re-excision BCS procedure (with negative BCS margins) and evaluation of the axillary lymph nodes (with recurrence 15 months after the original re-excision BCS procedure). No isolated local recurrences within the treated breast alone have been identified. Of the eight known deaths identified during the study period, two deaths are from among the three cases of documented recurrent breast cancer described above, five deaths are secondary to progression of other concurrent malignancies (including lung cancer in two cases, colon cancer in one case, lymphoma in one case, and leukemia in one case), and one death is from other (i.e. non-oncologic) causes.

## Discussion

The determination of the adequacy of BCS has long been debated among clinicians involved in the management of invasive breast cancer [8-17]. Central to this ongoing debate has been the definition of "adequate" or "negative" surgical resection margins, as well as the determination of what is the most optimal manner in which to assess the adequacy of the surgical resection margins. As illustrated in Table 12, the definition and determination of margin positivity during BCS for invasive breast cancer varies widely among all reported series [20-68]. From the endless number of discussions and commentaries on this topic within the medical literature [8-17], it is quite clear that there is absolutely no consensus among clinicians in regards to these important questions. And quite frankly, it is doubtful that any generalized consensus will ever be reached in the near future. Nevertheless, developing a standardized and reproducible methodology for the comprehensive and systematic assessment of surgical resection margins during BCS for invasive breast cancer while still limiting the volume of additional breast tissue excised remains an important issue to clinicians for most optimally assessing which individuals need further resection of the affected breast and for minimizing the long-term risks of local recurrence following BCS.

**Table 12:** Studies reporting margin positivity with breast-conserving surgery (BCS)*^,#^

Citation	Positive margin status (results and definition)
McCormick 1987 [20]	24.1% (26/108), defined as tumor at edge
Umpleby 1988 [21]	25.0% (13/52), defined as tumor in cavity bed specimens and not based on BCS specimen
England 1994 [22]	35.0% (28/80), defined as tumor < 1 mm from edge
MacMillan 1994 [23]	38.3% (101/264), defined as tumor in cavity bed specimens and not based on BCS specimen
Schnitt 1994 [24]	47.5% (86/181), defined as tumor at edge; 61.3% (111/181), defined as tumor ≤ 1 mm from edge
Beron 1996 [25]	51.9% (41/79), defined as tumor < 1 mm from edge
Gage 1996 [26]	38.5% (131/340), defined as tumor at edge, 54.4% (185/340), defined as tumor ≤ 1 mm from edge
MacMillan 1997 [27]	39.3% (118/300), defined as tumor in cavity bed specimens and not based on BCS specimen
Saarela 1997 [28]	14.5% (8/55), defined as tumor at edge
Weber 1997 [29]	15.0% (21/140), defined as tumor in cavity bed specimens and not based on BCS specimen
Beck 1998 [30]	27.1% (39/141), defined as tumor at edge
DiBiase 1998 [31]	19.0% (86/453), defined as tumor in cavity bed specimens and not based on BCS specimen
Taylor 1998 [32]	25.4% (68/268), defined as tumor in cavity bed specimens and not based on BCS specimen
Horiguchi 1999 [33]	22.4% (36/161), defined as tumor < 5 mm from edge
Malik 1999 [34]	36.8% (200/543), defined as tumor in cavity bed specimens and not based on BCS specimen
Papa 1999 [35]	29.1% (115/395), defined as tumor at edge
Park 2000 [36]	36.0% (192/533), defined as tumor at edge; 53.7% (286/533), defined as tumor ≤ 1 mm from edge
Gibson 2001 [37]	44.5% (243/546), defined as tumor at edge
Jenkinson 2001 [38]	18.8% (19/101), defined as tumor at edge
Moore 2001 [39]	15.7% (8/51), defined as tumor at edge
Swanson 2002 [40]	32.7% (85/260), defined as tumor at edge; 45.0% (117/260), defined as tumor < 2 mm from edge
Mai 2003 [41]	25.8% (16/62), defined as tumor < 1 mm from edge
Chagpur 2004 [42]	12.4% (329/2658), defined as tumor at edge
Keskek 2004 [43]	39.6% (120/303), defined as tumor ≤ 2 mm from edge
Miller 2004 [44]	18.4% (26/141), defined as tumor at edge
Fleming 2004 [45]	9.1% (20/220), defined as tumor < 5 mm from edge
Balch 2005 [46]	25.1% (64/255), defined as tumor < 2 mm from edge
Cao 2005 [47]	81.7% (103/126), defined as tumor ≤ 2 mm from edge
Cendán 2005 [48]	44.3% (43/97), defined as tumor in cavity bed specimens and not based on BCS specimen
Dooley 2005 [49]	11.4% (25/220), defined as tumor ≤ 1 mm from edge
Nadeem 2005 [50]	30.0% (39/130), defined a tumor < 1 mm from edge
Aziz 2006 [51]	14.3% (205/1430), defined as tumor at edge
Dillon 2006 [52]	34.5% (211/612), defined as tumor < 5 mm from edge
Huston 2006 [53]	61.4% (105/171), defined as tumor < 2 mm from edge
Janes 2006 [54]	44.2% (96/217), defined as tumor < 5 mm from edge
Méndez 2006 [55]	64.6% (115/178), defined as tumor ≤ 2 mm from edge
Cabioglu 2007 [56]	13.6% (27/200), defined as tumor at edge; 32.7% (65/200), defined as tumor ≤ 2 mm from edge
Kotwall 2007 [57]	52.6% (306/582), defined as tumor at edge
Smitt 2007 [58]	43.5% (172/395), defined as tumor at edge
Wright 2007 [59]	16.0% (42/263), defined as tumor at edge; 41.8% (110/263), defined as tumor ≤ 1 mm from edge
Dillon 2008 [60]	19.9% (56/281), defined as tumor < 2 mm from edge; 32.0% (90/281), defined as tumor < 5 mm from edge
Jacobson 2008 [61]	66.4% (83/125), defined as tumor ≤ 2 mm from edge
Schiller 2008 [62]	17.0% (124/730), defined as tumor at edge; 34.9% (255/730), defined as tumor < 1 mm from edge
Soucy 2008 [63]	18.4% (88/478), defined as tumor at edge
Lovrics 2008 [64]	19.6% (65/332), defined as tumor at edge
Sabel 2009 [65]	18.2% (173/948), defined as tumor at edge; 32.0% (303/948), defined as tumor ≤ 2 mm from edge
Tengher-Barna 2009 [66]	43.9% (47/107), defined as tumor ≤ 3 mm from edge
Munhoz 2009 [67]	28.8% (63/218), no definition of margin positivity given
Hewes 2009 [68]	20.5% (196/957), defined a tumor < 1 mm from edge
Povoski 2009	6.4% (13/204), defined as tumor at edge, 10.8% (22/204), defined as tumor ≤ 1 mm from edge

BCS, breast-conserving surgery

* This table excludes any previous studies that included in their analyses a significant percentage of patients with an "unknown" or "indeterminate" margin status in comparison to the reported percentage of patients with a positive margin status since this "unknown" or "indeterminate" variable would not allow for an accurate determination of the percentage of patients with margin positivity.

^# ^Some of these previous studies not only included individual undergoing a definitive BCS procedure, but also individuals undergoing only a diagnostic surgical excisional breast biopsy. In many such cases, this subtle distinction was not well articulated and likely accounted for their relatively high reported margin positivity rates.

In our current series, 6.4% (13/204) of patients had positive BCS specimen margins (defined as tumor cells at the inked edge of the BCS specimen) and 4.4% (9/204) of patients had close margins (defined as tumor cells within 1 mm or less of the inked edge but not at the inked edge of the BCS specimen). Based on a 1 mm or less criteria for the concern of a compromised surgical margin of resection at the time of a BCS procedure, a total of 10.8% (22/204) of patients were counseled about the potential clinical significance and implications of these findings. Our results, as compared to the multitude of other reported series showed in Table 12[20-68], are very respectable and are reflective of both the adherence to appropriate patient selection for BCS that was used by the surgeon performing these BCS procedures as well as the standardized rationale and technique that was used by the surgeon for the determination of the extent of tissue resection during each BCS procedure. Additionally, 11.8% (n = 24) of all 204 patients had at least one re-resection margin containing additional invasive carcinoma or DCIS, independent of the status of the BCS specimen margins. This included 7.1% (n = 13) of the 182 patients with negative BCS specimen margins (defined as no tumor cells seen within 1 mm or less of the inked edge of the BCS specimen) who had at least one re-resection margin containing additional invasive carcinoma or DCIS. Therefore, of the total of 24 patients with at least one re-resection margin containing additional invasive carcinoma or DCIS, 13 (54.2%, P < 0.001) represented individuals that had additional adjacent invasive carcinoma or DCIS that was not recognized by a standard BCS procedure alone, based upon the findings of negative BCS specimen margins (Table 9). Of a similarly related note regarding disconcordance, even when one or more of the BCS specimen margins were positive/close, 6 of 11 (54.5%) of such patients that also had at least one positive re-resection margin had some degree of disconcordance between the positive/close BCS specimen margin status and the positive re-resection margin status (Table 10), thus further recognizing the failure of surgical resection margin assessment that is solely based upon only evaluating the BCS specimen margins themselves.

The pattern of having disconcordant findings, in which there are negative margins on the BCS specimen itself despite the finding of further disease within additionally re-excised tissue from within the BCS resection bed cavity, has been previously reported by multiple other authors [22,30,38,43,47,53,66,68]. In 1994, England et al [22] reported finding positive disease in the "tumour bed biopsy" re-excision specimens in 9.6% (5/52) of patients with initial negative "wide local excision" margins. In 1998, Beck et al [30] reported finding positive disease in the "entire cavity wall" re-excision specimen in 13.3% (14/105) of patients with initial negative "wide local excision" margins. In 2001, Jenkinson et al [38] reported finding positive disease in the "cavity bed biopsy" re-excision specimens in 6.0% (5/83) of patients with initial negative "segmental mastectomy" margins. In 2004, Keskek et al [43] reported finding positive disease in "complete cavity margin excision" specimens in 4.3% (13/303) of patients with initial negative margins on the "initial tumour excision". In 2005, Cao et al [47] reported finding positive disease in the "cavity margin" re-excision specimens in 8.7% (2/23) of patients with initial negative "lumpectomy" margins. In 2006, Huston et al [53] reported finding positive disease in "additional margin" specimens in 6.4% (3/26) of patients with initial negative "lumpectomy" margins. In 2009, Tengher-Barna et al [66] reported finding positive disease in "cavity margin" specimens in 10.3% (11/107) of patients with initial negative "lumpectomy" margins. Finally, in 2009, Hewes et al [68] reported finding positive disease in "cavity biopsy" specimens in 8.6% (82/957) of patients with initial negative "wide local excision" margins.

In this specific regard, the disconcordance of having negative BCS specimen margins and positive re-resection margins (Table 9), as well as the high degree of disconcordance shown in cases having both positive/close BCS specimen margins and positive resection margins (Table 10), appears to signify that a substantial percentage of failed BCS procedures may result, not necessarily from unrecognized positive or close margins on the BCS specimen itself, but rather from the presence of adjacent multifocal disease within the same quadrant of the affected breast upon which the BCS procedure was performed. Beck et al [30] described this phenomenon as a "discontinuous growth pattern commonly seen around primary tumours". Other investigators examining BCS have also described this phenomenon [69,70]. Likewise, Holland et al [71] previously evaluate and discussed this phenomenon in a retrospective review of mastectomy specimens from 1980 to 1982 in which 121 of 282 (43%) mastectomy specimens from patients that would have theoretically been candidates for BCS based on standard clinical and radiographic criteria for that time period had additional disease within the affected breast that was greater than 2 cm from the known breast cancer. Despite the plausibility of our above explanation, which is clearly supported by the work of other investigators [30,69-71], that adjacent multifocal disease within the same quadrant of the affected breast may contribute to the occurrence of many failed BCS procedures, we can not rule out that some failed BCS procedures may result from suboptimal surgical resection margin assessment techniques that fail to recognized positive or close margins on the BCS specimen itself. Nevertheless, in regards to the issue of the potential presence of adjacent multifocal disease within the same quadrant of the affected breast upon which the BCS procedure is being performed, obtaining nine standardized re-resection margin specimens, as proposed in our current study, allows one to systematically and methodically assess the adjacent breast tissue for multifocality within the same quadrant of the affected breast and may allow one to select out those individuals who would be better suited for completion mastectomy who had additional multifocal disease within the same quadrant that would have otherwise been unrecognized by traditional resectional techniques for BCS that did not routinely utilize some sort of re-resection margin technique.

This line of reasoning does not disagree with the well-held contention that most breast cancers recurrences within the breast after BCS occur within the same quadrant of the affected breast and not within a different quadrant of the affected breast [30,70,72-76]. Instead, our line of reasoning agrees with this well-held contention since it is our belief that such recurrences within the affected breast represent a failure of BCS, in the sense that there is a failure to recognize adjacent multifocality located adjacent to the primary tumor mass rather than a failure of the traditional resectional technique itself to remove the primary tumor mass in its entirety from the affected breast. Thus, by using the nine standardized re-resection margin technique to identify as many such cases that are not realistically amendable to BCS and that instead are truly in need of a completion mastectomy to successfully remove all multifocality of the breast cancer, it is possible that such an approach could ultimately translate into less local breast recurrences from successful BCS procedures (i.e., better patient selection for those who can be successfully managed by BCS and better patient selection for those who should be best managed by completion mastectomy).

Multiple authors have previously described various methodologies for attempting to recognize residual disease within the walls of the BCS resection bed cavity of the affected breast that is not otherwise well appreciated by the current generally accepted technique of only evaluating of the margins of the BCS specimen alone [21-23,27,29-32,34,38,43,47,48,53,54,61,66,68,77,78]. Specific to this discussion are those studies that have focus specifically upon methods of further removal of tissue from the walls of the BCS resection bed cavity after the removal of the BCS specimen. Some of those authors have described a "selective cavity margin sampling" technique [21,22,29,31,32,38,47,48,53,54,61,66,68,77,78], while others have described "complete cavity margin re-excision" technique [23,27,30,34,43]. Such a discussion will not include those studies specifically addressing methodologies for image-guided wire placement, intraoperative ultrasound guidance, radioguided techniques for guiding the resection of the BCS specimen, or intraoperative detection of electrical property changes or optical property changes within the BCS resection bed cavity.

As is shown in Table 13, those authors that have described a "selective cavity margin sampling" technique show great variability in their sampling methodology [21,22,29,31,32,38,47,48,53,54,61,66,68,77,78]. These various methods of "selective cavity margin sampling" [21,22,29,31,32,38,47,48,53,54,61,66,68,77,78] were all very effective at demonstrating additional disease within the affected breast that would have been otherwise unrecognized by the current generally accepted technique of evaluation of the margin status of the BCS specimen alone. Additionally, a collective conclusion drawn by each of these studies that specifically evaluated the overall BCS margin status based upon both the "selective cavity margin sampling" specimens and the BCS specimen itself was that such a combined approach also effectively reduced the need for a subsequent re-excision procedure to the affected breast at a later time as compared to the reliance only upon the current generally accepted technique of evaluation of the margin status of the BCS specimen alone, specifically for those individuals with involved BCS specimen margins but uninvolved "selective cavity margin sampling" specimens [47,53,61,66,68]. In contrast to the various "selective cavity margin sampling" techniques shown in Table 13 which generally sample up to six areas within the BCS resection bed cavity [21,22,29,31,32,38,47,48,53,54,61,66,68,77,78], our technique of nine standardized re-resection margins attempts to further maximize the number of surfaces of the BCS resection bed cavity that are sampled and evaluated, while still limiting the total amount of additional breast tissue removed.

**Table 13:** Studies reporting "selective cavity margin sampling" techniques for breast-conserving surgery (BCS)

Citation	Methodology for "selective cavity margin sampling"
Umpleby 1988 [21]	5 "cavity biopsies" from the superior, inferior, lateral, medial, and deep margins of the cavity wall that were evaluated by permanent histopathology, but for which no data was reported on the margin status of the BCS specimen
England 1994 [22]	5 "tumour bed biopsies" from the superior, inferior, medial, lateral, and base of cavity that were evaluated by permanent histopathology along with evaluation of the margins of the BCS specimen
Weber 1997 [29]	5 "tumor cavity biopsies" from the medial, lateral, superior, inferior, and deep aspects of the lumpectomy cavity that were evaluated by frozen section and permanent histopathology, but for which no data was reported on the margin status of the BCS specimen
Dibiase 1998 [31]	5 "tumor cavity shaved biopsies" from the medial, lateral, superior, inferior, and base of cavity that were evaluated by permanent histopathology, but for which no data was reported on the margin status of the BCS specimen
Taylor 1998 [32]	4 "bed biopsies" from each of the 4 quadrants of the post-resection bed that were evaluated by permanent histopathology, but for which no data was reported on the margin status of the BCS specimen
Jenkinson 2001 [38]	4 "tumour bed biopsies" from each of the 4 quadrants of the post-resection bed that were evaluated by permanent histopathology along with evaluation of the margins of the BCS specimen
Cao 2005 [47]	4 to 6 "cavity margins" from either the superior, inferior, medial, lateral, anterior, and/or posterior aspects of the residual cavity that were evaluated by permanent histopathology along with evaluation of the margins of the BCS specimen
Cendán 2005 [48]	5 to 6 "cavity margins" from either the lateral, medial, inferior, superior, deep, and/or superficial aspects of the lumpectomy cavity that were evaluated by frozen section and permanent histopathology, but for which no data was reported on the margin status of the BCS specimen
Huston 2006 [53]	Comparative study of taking 4 to 6 "additional margins" versus 1 to 3 "additional margins" versus no "additional margins" (with the specific excision locations not designated for those "additional margins") that were evaluated by permanent histopathology along with evaluation of the margins of the BCS specimen
Janes 2006 [54]	2 standardized "cavity shaves" from the superior and inferior aspects of the residual cavity that were evaluated by permanent histopathology along with evaluation of the margins of the BCS specimen
Olson 2007 [77]	3 to 6 "cavity margin biopsies" from the walls of the BCS cavity that were evaluated by frozen section and permanent histopathology, but for which no data was reported on the margin status of the BCS specimen
Jacobson 2008 [61]	4 to 6 "shaved margins" from either the superior, inferior, medial, lateral, anterior, and/or posterior aspect of the lumpectomy cavity that were evaluated by permanent histopathology along with evaluation of the margins of the BCS specimen
Marudanayagam 2008 [78]	Up to 4 "cavity shavings" from either the superior, inferior, medial, and/or lateral aspects of the cavity that were evaluated by permanent histopathology, but for which no data was reported on the margin status of the BCS specimen
Tengher-Barna 2009 [66]	4 "cavity margins" from the lateral, medial, superior, and inferior of the lumpectomy cavity that were evaluated by permanent histopathology along with evaluation of the margins of the BCS specimen
Hewes 2009[68]	4 "cavity biopsies" from the 4 quadrants of the residual cavity that were evaluated by permanent histopathology along with evaluation of the margins of the BCS specimen

BCS, breast-conserving surgery

Several authors have described a technique of "complete cavity margin re-excision" for comprehensive evaluation of the surgical resection margins at the time of BCS [23,27,30,34,43]. Although the "complete cavity margin re-excision" technique seems ideal for achieving the most thorough assessment of the surgical resection margins, it significantly increases the overall volume of tissue removed from the affected breast, it is limited by breast size, and it frequently leads to very suboptimal cosmetic results. MacMillan and colleagues [23,27,24] described a method of "complete cavity margin re-excision" in which the entire cavity wall was excised as a single continuous shell of tissue that was evaluated by permanent histopathology, but for which no data was reported on the margin status of the BCS specimen. Beck et al [30] described a method of "complete cavity margin re-excision" in which the entire cavity wall was excised as a single continuous ring of tissue that was evaluated by permanent histopathology along with evaluation of the margins of the BCS specimen. Keskek et al [43] described a method of "complete cavity margin re-excision" in which the entire cavity wall was excised as four separate specimens (designated superior, lateral, inferior, and medial) that were evaluated by permanent histopathology along with evaluation of the margins of the BCS specimen. These various methods of "complete cavity margin re-excision" [23,27,30,34,43] were all very effective at demonstrating additional disease within the affected breast that would have been otherwise unrecognized by the current generally accepted technique of evaluation of the margin status of the BCS specimen alone. However, the major drawback to the "complete cavity margin re-excision" technique is that the amount of tissue removed by performing the "complete cavity margin re-excision" can essentially equal the amount of tissue removed as the BCS specimen itself [30], thus doubling the total size the BCS resection bed cavity within the affected breast and thus subsequent negatively impacting on the cosmetic outcome of the BCS procedure. In contrast to the "complete cavity margin re-excision" techniques [23,27,30,34,43], our technique of nine standardized re-resection margins minimizes amount of additional tissue excised from the affected breast for accurate assessment of the presence or absence of further disease around the BCS specimen (i.e., representing only 26.8% of the volume of the BCS specimen and 32.6% of the surface area of the BCS specimen) while still maximizing the number of different surfaces within the BCS resection bed cavity that are sampled (i.e., nine standardized re-resection margins).

Several investigators have addressed and debated the efficacy of the technique of performing gross intraoperative examination of the BCS margins and selective immediate intraoperative re-excision of any given BCS margin that is thought to represent a suspicious or compromised margin [45,46,56,63] as a mechanism to reduce the need for a subsequent second re-excision procedure. We did not utilize this methodology in our series of 204 BCS procedures. As with any proposed technique, no universal consensus has been reach on the efficacy of performing gross intraoperative examination of the BCS margins and selective immediate intraoperative re-excision of BCS margins that are thought to represent suspicious or compromised margins. Although this assessment technique for surgical resection margins varies to some degree between reported series, it is primarily based upon the gross visual inspection of the intact BCS specimen as well as gross visual inspection the cut surface of inked, non-fixed, sectioned BCS specimen at the time of BCS. In this regard, it is quite evident that one faction strongly favors its use [45] while another faction does not [46].

Fleming et al [45] have previously reported that "intraoperative macroscopic margin assessment" is an effective technique for reducing the number of second operations in patients undergoing BCS for primary invasive breast cancer. They demonstrated that only 9.1% (20/220) of patients required a second operation when "intraoperative macroscopic margin assessment" was utilized as compared to 21.4% (47/220) of patients would have required a second operation if "intraoperative macroscopic margin assessment" had not been utilized. However, interestingly, in their study, only 19.8% (16/81) of patients that had macroscopically suspicious or compromised margins (as they defined as suspected tumor grossly/macroscopically within 10 mm of any given gross/macroscopic margin) at the time of the initial BCS surgery in which they then performed further selective immediate intraoperative re-excision of BCS margins actually had a final histologic-confirmed positive margin status on the original BCS specimen (as they defined as invasive carcinoma or DCIS at a distance of less than 5 mm from the microscopic margin on permanent histopathology). In such series as Fleming et al [45], as well as others [33,45,52,54,79,80] that routinely advocate very wide microscopic margins of at least 5 mm on permanent histopathology to define a margin status as negative, the utilization of our proposed nine standardized re-resection margins technique for the BCS resection bed cavity would have allowed those surgeon to consider far less extreme criteria for a negative margin status on the BCS specimen itself and may have provided them with more confidence that complete tumor excision had been successfully accomplished.

In contract, Balch et al [46] have previously reported that "intraoperative gross examination" of surgical resection margins was not particularly useful for patients undergoing BCS for breast cancer. In their study, they reported that only 32.6% (46/141) of patients that had grossly suspicious or compromised margins (as they defined as suspected tumor grossly/macroscopically within 5 mm of any given gross/macroscopic margin) at the time of the initial BCS actually had final histologically positive margins on the original BCS specimen (as they defined as invasive carcinoma or DCIS at a distance of within 2 mm from the microscopic margin on permanent histopathology). Additionally, they reported that "intraoperative gross examination" of surgical resection margins did not accurately reflect the margin status since 28.1% (18/64) of patients with final histologically positive margins (as they defined as invasive carcinoma or DCIS at a distance of within 2 mm from the microscopic margin on permanent histopathology) were initially thought to have grossly tumor-free margins (as they defined as no suspected tumor grossly/macroscopically within 5 mm of any given gross/macroscopic margin). Therefore, they recommended consideration of other potential techniques for intraoperative evaluation of surgical resection margins to attempt to reduce the need for a subsequent re-excision in patients undergoing BCS.

For the assessment of surgical resection margins on the BCS specimen itself, it is pertinent for us to discuss and contrast the use of the "tangential shaved margins" technique [9,55,59,81] versus that of the currently more widely accepted "perpendicular margins" technique (i.e., "radial margins" technique). In our series, the assessment of the surgical resection margins on the BCS specimen itself was performed by obtaining perpendicular sections along the entire long axis length of the BCS specimen, thus recognizably representing a rather limited sampling of the total margin surface of the BCS specimen. With the "perpendicular margins" technique on the BCS specimen itself, it is only when tumor cells are present at the inked edge of a particular perpendicular section being microscopically examined that the surgical resection margin is considered positive. The major advantage of this "perpendicular margins" method is that the relationship and accurate measurement of the distance between the tumor and the surgical resection margin surface of the BCS specimen itself can be microscopically evaluated. Alternatively, surgical resection margins on the BCS specimen can be assessed by utilizing the so called "tangential shaved margins" technique [9,55,59,81]. In this method, the surface of the BCS specimen itself is either shaved [55,59,81] or peeled [9], and the resulting thin tissue sections are placed flat in cassettes for embedding, resulting in tangentially sectioned surfaces for microscopic margin assessment. With the "tangential shaved margins" technique, the presence of any tumor cells within an examined section is grounds for interpretation as a positive surgical resection margin status. Because one is actually tangential sectioning into the periphery of the BCS specimen itself with the use of the "tangential shaved margins" technique, a measurement of the actual distance between the tumor and the surgical resection margin surface of the BCS specimen itself can not be accurately determined. Nevertheless, major advantage of this "tangential shaved margins" technique is that the total area of the general periphery of the BCS specimen itself that is microscopically examined is significantly greater. Therefore, the use of one or the other of these two sampling techniques, when applied to the BCS specimen itself, represent a compromise between allowing direct microscopic visualization of the relationship between the tumor and the surgical resection margins and providing variable degrees of the total breast tissue "surface" area that is microscopically examined for surgical margin assessment.

It is evident that the use of the "tangential shaved margins" technique on the BCS specimen itself [59] results in a significantly higher rate of "margin positivity" than what is generally seen by the more widely accepted technique of "perpendicular margins" for assessment of surgical resection margins on the BCS specimen itself. Since, within our current study methodology, we did not compare the efficacy of the "tangential shaved margins" technique versus that of the "perpendicular margins" technique for assessment of surgical resection margins on the BCS specimen itself, it is difficult for us to critically debate the pros and cons of the use of the "tangential shaved margins" technique for assessment of surgical resection margins on the BCS specimen itself. Nevertheless, we, as well as others [59,81,82], do recognize that, in general, the concept of a "tangential shaved margins" technique is an obvious and important step toward improving the currently accepted and utilized methodology for the assessment of surgical resection margins on the BCS specimen alone. This is simply because the "tangential shaved margins" technique on the BCS specimen itself dramatically increases the total breast tissue "surface" area of the BCS specimen that is available for surgical resection margin assessment, while, on the other hand, the "perpendicular margins" technique on the BCS specimen itself can be intrinsically less sensitive secondary to the fact that it provides surgical resection margin assessment of only a much smaller fraction of the surface area of the BCS specimen itself. Yet, at the same time, we also believe that the use of the "tangential shaved margins" technique on the BCS specimen itself is not necessarily a true reflection of what is going on directly at the surface of the BCS specimen itself, since it represent the action of sectioning directly into the peripheral tissues of the BCS specimen, a process which compromises the integrity of the periphery of the BCS specimen and is potentially fraught with many technical issues [82]. In our estimation, a "selective cavity margin sampling" technique for the BCS resection bed cavity, which essential represents an equivalent to the "tangential shaved margins" technique on the BCS specimen itself and which allows for a greater percentage of the surface area of breast tissue directly adjacent to the periphery of the BCS specimen itself (i.e., the BCS resection bed cavity) to be available for surgical resection margin assessment (but without compromising the integrity of the BCS specimen itself) is a better solution. Therefore, as is the basis of our current paper, when our nine standardized re-resection margin technique is used as the "selective cavity margin sampling" technique for the BCS resection bed cavity and is combined with the generally accepted "perpendicular margins" technique for assessment of surgical resection margins on the BCS specimen itself, we believe that we can achieved the "best of both worlds" with regards to the development of a standardized and reproducible methodology for the comprehensive and systematic assessment of surgical resection margins during BCS. Such a combined approach gives rise to the concept of optimizing the determination of region clearance of tumor burden within the affected breast by taking into account both the potential for microscopically recognized or unrecognized compromise of the surgical resection margins of the BCS specimen itself, as well as the potential for unrecognized multifocality of disease within the same quadrant of the affected breast.

Multiple authors [16,47,49,68,83] have previously discussed the technical issues related to the generation of false positive margins on BCS specimens. General handing of the BCS specimen (that can take place either before or after normal specimen processing and inking that occurs in the pathology department), as well as specimen compression at the time of specimen mammography, can significantly impact upon the creation of artificially close or false positive margins. Due to the friable and lobular nature of the surfaces of both the fibrofatty tissue component and mammary tissue component comprising the freshly excised BCS specimen, any compression to the freshly excised BCS specimen can create artificial crevices, tissue thinning, and reorientation of the overlying fibrofatty tissue and mammary tissue covering the actual tumor surface. This can result in decreasing the actual distance between the surface of the freshly excised BCS specimen and the surface of the tumor, as well as result in a change in the orientation of which surface of the freshly excised BCS specimen actually represents the true, closest margin. The subsequent application of ink to the friable and lobular surfaces of the freshly excised BCS specimen, and as accentuated by the previous above described effect of specimen compression, can result in the ingress of ink into these artificial crevices and potential spaces located in between the uneven, friable, and lobular surfaces of the freshly excised BCS specimen that ultimately allows for tracking and deposition of ink into locations more closely approximating the surface of the tumor than are actually representative of the true distance from the intended margin surface of the freshly excised BCS specimen to the actual tumor surface. Additionally, the utilization of overgenerous amounts of ink, as well as the addition of any excess force during the process of ink application to the freshly excised BCS specimen and during the process of removal of excess ink (i.e., "blotting dry") from the freshly excised BCS specimen can further accentuate the ingress of ink into potential spaces that are not representative of the intended margin surface of the freshly excised BCS specimen. Taken together, all these above describe variables may lead to the generation of artificially elevated false positive margin rates and may be a major contributing factor in the alarmingly high rate of margin positivity reported in many of the studies shown in Table 12[20-68]. Better education of those individuals that are directly involved in specimen transport, specimen mammographic evaluation, and specimen processing within the pathology department may help to reduce these potentially preventable causes of an artificially elevated rate of margin positivity on BCS specimens.

When one reflects on the overall alarmingly high rate of margin positivity reported by many previous series in the literature and which is clearly illustrated within Table 12[20-68], the validity of such data that is thought to represent homogenous populations of patients undergoing definitive BCS then comes into question. Clearly, some of these previous reported series not only included individual undergoing a definitive BCS procedure, but also individuals undergoing only a diagnostic surgical excisional breast biopsy. This subtle distinction was generally not well articulated by the authors of such papers, and this factor, in many cases, may have accounted for their relatively high reported margin positivity rates. Two recent studies have clearly addressed this issue [58,64]. Lovrics et al [64] nicely illustrated the significance of having a confirmed preoperative diagnosis of invasive breast cancer on margin positivity with BCS. They showed that 19.6% (65/332) of patients had positive margins (defined by tumor at cut edge) on BCS specimens when there was a confirmed preoperative diagnosis of invasive breast cancer while 26.0% (127/489) of patients had positive margins on BCS specimens when they included those patients without a confirmed preoperative diagnosis of invasive breast cancer. This translated into 42.2% (62/147) of patients had positive margins on BCS specimens when there was specifically no confirmed preoperative diagnosis of invasive breast cancer. This difference was highly significant on both univariate and multivariate analyses (P < 0.001). Similarly, Smitt et al [58] showed that 71.3% (234/328) of those without a preoperative diagnosis of invasive breast cancer had positive/close margins (defined by tumor within 2 mm from the inked edge) on the BCS specimen in comparison to only 47.8% (32/67) of those with a preoperative diagnosis of invasive breast cancer on core/FNA had positive/close margins on the BCS specimen (P < 0.0001).

Finally, in our current series, the finding of DCIS in conjunction with invasive carcinoma was the only variable that we evaluated that was associated with a "positive final margin status" on both univariate and multivariate analyses (Table 11). This association of having a "positive final margin status" when DCIS is found in conjunction with invasive carcinoma has been previously described in the literature [41,44,84]. Such an association is thought to be an important predictor of finding residual disease within the affected breast at the time of re-excision [25,85-87], as well as an important predictor of long-term recurrence of disease within the affected breast [88-96]. However, a more in-depth characterization of this association can not be gleaned from our current results since we did not attempt to distinguish the simple finding of associated DCIS from that of the more intricate definition of finding of an extensive intraductal component associated with an invasive breast cancer.

## Conclusion

In our current report, we have presented a standardized and reproducible methodology for comprehensive and systematic assessment of surgical resection margins during BCS. Such a combined approach gives rise to the concept of determination of region clearance of tumor burden within the affected breast by taking into account both the potential for microscopically recognized or unrecognized compromise of the surgical resection margins of the BCS specimen itself, as well as the potential for unrecognized multifocality of disease within the same quadrant of the affected breast. Our methodology accurately assesses the adequacy of surgical resection margins for determination of which individuals may need further resection to the affected breast in order to minimize the potential risk of local recurrence following BCS, while attempting to limit the volume of additional breast tissue excised at the time of BCS. Likewise, our methodology is useful in determining which individuals are not realistically amendable to BCS and that are instead in need of a completion mastectomy to successfully remove multifocal disease that would have otherwise been unrecognized by traditional resectional techniques for BCS that do not routinely utilize some sort of re-resection margin technique.

## Competing interests

The authors declare that they have no competing interests.

## Authors' contributions

**SPP **was the surgeon who performed all the BCS procedures. He designed the current study, collected the data, and performed all data analyses. He organized, wrote, and edited all aspects of this manuscript. **REJ **was the pathologist who was involved in reading the original histopathology for many of the cases contained within this series, was responsible for reevaluating those cases with "close" margins, and was involved in the writing and editing of the manuscript. **WPW **was the pathologist who was involved in reading the original histopathology for some of the cases contained within this series and was involved in the writing and editing of the manuscript. **RXX **was involved in the technical aspects of the data analyses and was involved in the writing and editing of the manuscript. All of the authors have read and approved the final version of this manuscript.

## Pre-publication history

The pre-publication history for this paper can be accessed here:

http://www.biomedcentral.com/1471-2407/9/254/prepub
